# Replicated, urban-driven exposure to metallic trace elements in two passerines

**DOI:** 10.1038/s41598-021-99329-2

**Published:** 2021-10-04

**Authors:** Marion Chatelain, Arnaud Da Silva, Marta Celej, Eliza Kurek, Ewa Bulska, Michela Corsini, Marta Szulkin

**Affiliations:** 1 Centre of New Technologies, University of Warsaw, 02-097 Warsaw, Poland; 2 Department of Zoology, University of Innsbruck, Technikerstraße 25, 6020 Innsbruck, Austria; 3 Faculty of Chemistry, Biological and Chemical Research Centre, University of Warsaw, 02-089 Warsaw, Poland

**Keywords:** Evolutionary ecology, Urban ecology, Ecology, Zoology

## Abstract

While there are increasing examples of phenotypic and genotypic differences between urban and non-urban populations of plants and animals, few studies identified the mechanisms explaining those dissimilarities. The characterization of the urban landscape, which can only be achieved by measuring variability in relevant environmental factors within and between cities, is a keystone prerequisite to understand the effects of urbanization on wildlife. Here, we measured variation in bird exposure to metal pollution within 8 replicated urbanization gradients and within 2 flagship bird species in urban evolutionary ecology: the blue tit (*Cyanistes caeruleus*) and the great tit (*Parus major*). We report on a highly significant, positive linear relationship between the magnitude of urbanization—inferred as either tree cover, impervious surface cover, or an urbanization score computed from several environmental variables, and copper, zinc and lead concentrations in bird feathers. The reverse relationship was measured in the case of mercury, while cadmium and arsenic did not vary in response to the urbanization level. This result, replicated across multiple cities and two passerine species, strongly suggests that copper, zinc, lead and mercury pollution is likely to trigger the emergence of parallel responses at the phenotypic and/or genotypic level between urban environments worldwide.

## Introduction

There is increasing evidence that urbanization is associated with modifications to plant and animal communities and populations^1–9^. For instance, birds inhabiting urbanized environments tend to suffer from lower reproductive outputs^4^ and higher physiological costs (e.g., higher oxidative stress^10^ and shorter telomeres^11^). While chemical, light and noise pollution, as well as human presence and altered food availability and quality are often suggested as the main potential drivers of those phenotypic changes in urban areas^12–14^, few studies actually identified the environmental factors responsible for such modifications^15,16^*.* One of the reasons is that the majority of studies focussing on wildlife ecology and biology in the urban space uses an extremely simplified urban ecology framework that often lacks (i) adequate replication stemming from a comparison of multiple cities and (ii) knowledge stemming from multiple and contrasted urban habitats contributing to the urban mosaic. While awareness of these limitations is recently growing^17,18^, all too often urban ecology inference focuses on the phenotypes of individuals caught within one location in a city (often urban parks) and outside of this same city (usually in forests adjacent to the city)^19^. Thus, such study design ignores the diversity of environments within and between urbanized areas^20^, which prevents from (i) establishing firm conclusions about the effects of urbanization per se on wildlife, (ii) disentangling the effect of the different abiotic and biotic environmental factors that vary in response to urbanization^21^, and (iii) drawing universal conclusions about the impact of urbanization on the biology of wild organisms at a continental or global scale. For this reason, recent reviews in urban ecology urge future research to focus on replicated and continuous gradients of disturbance^2,3,14,19^. Indeed, measuring how potential environmental stress factors vary within the urban mosaics across multiple cities is without a doubt a prerequisite to further understand the effects of urbanization on wildlife.

Metallic/metalloid trace elements (MTEs; e.g., lead, cadmium, copper) are a major class of pollutants that may have lethal and sublethal effects on organisms^22^. In birds, individuals nesting close to metallurgic smelters show reduced reproductive outputs^23–25^. While less documented, similar trends were measured in bird exposed to urban MTE pollution^16,26^. MTEs are mainly emitted by anthropogenic activities^27^. In urban environments, road traffic, residential heating, coal burning, and industry are the main sources of MTE pollution^28^. Literature on bird exposure to MTEs is abundant, although only a minority of studies focussed on urban MTE pollution^29^, and all of those studies but three^15,16,30^ compared MTE concentrations in individuals using the coarse dichotomy of urban versus non-urban areas^31–40^. Those studies measured MTEs in feathers, blood, liver, kidney, bones or eggshell. While most studies reported higher levels of lead at urban sites than at non-urban sites, the impact of urbanization on other MTEs varied^15,16,30–40^. All in all, while MTE pollution might be a significant driver of phenotypic and/or genotypic changes triggered by urbanization, we currently lack knowledge on MTE pollution levels within complex urban–rural gradients.

To fill in this gap, we measured 6 of the most common MTE pollutants (i.e. copper, zinc, lead, cadmium, arsenic and mercury) in the feathers of 179 males of blue tits (*Cyanistes caeruleus*) and great tits (*Parus major*). Importantly, feathers are one of the often used non-invasive material for the biomonitoring of MTE exposure in birds^16,29,41^. Birds have been sampled in a continuous gradient of urbanization replicated across 8 cities (i.e. densely populated areas with more than 50,000 inhabitants^42^) in Poland. Here, we define urbanization using high-resolution environmental data. This quantitative approach was compared with a qualitative approach where sampling sites were sorted into 5 habitat categories. Thanks to this unique study design, we address whether bird exposure to MTEs, assessed in two passerine species, varies consistently, linearly and in a replicated fashion along multiple urbanization gradients.

## Methods

### Bird sampling

**Figure 1:**
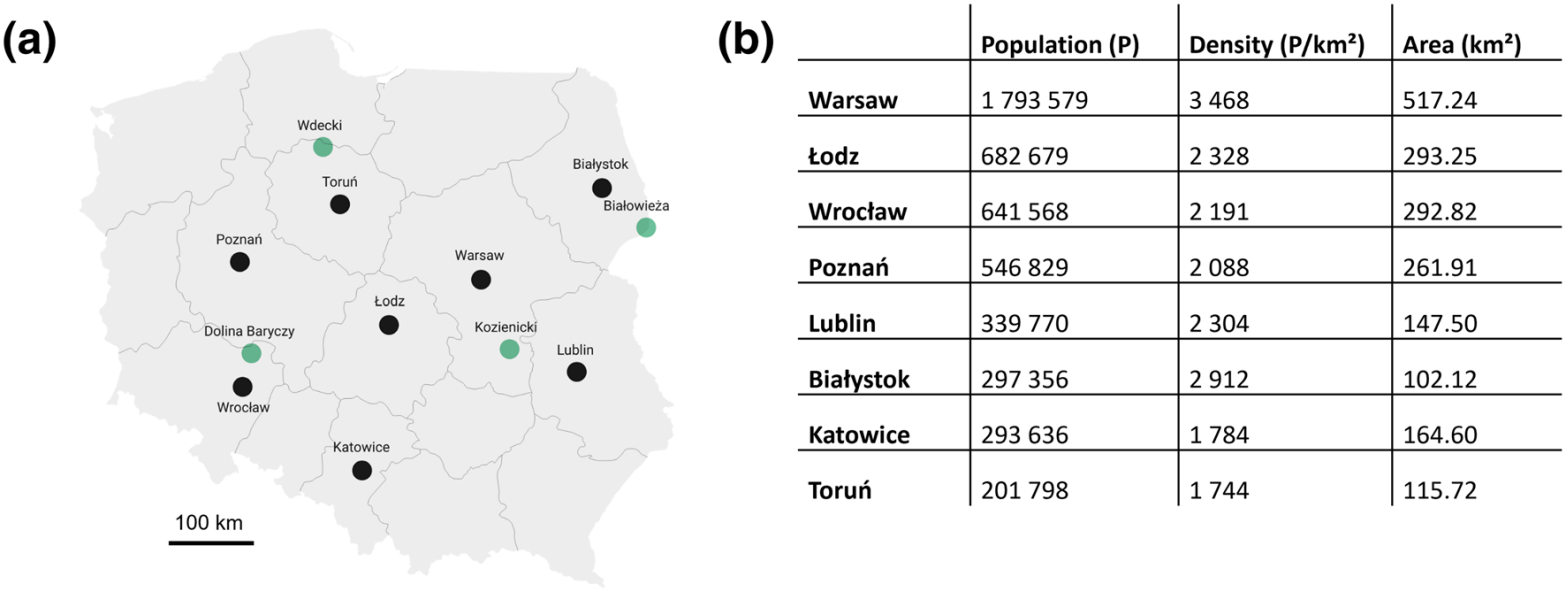
Sampling points—(**a**) Map of Poland highlighting the 8 cities (in black) and 4 protected forests (in green) where blue tits and great tits were sampled. (**b**) For the 8 cities, we detail human population size, population density and urban area size as defined by administrative borders (data as of 2019 from Central Statistics Poland—GUS).

Two passerine bird species, the Blue Tit (*Cyanistes caeruleus*) and the Great Tit (*Parus major*) were caught using mist-nets in 8 cities (i.e. Warsaw, Łódż, Wrocław, Poznań, Lublin, Białystok, Katowice and Toruń), 8 suburban forests (adjacent to the 8 cities listed above) and 4 large complexes of protected forests (i.e. Bialowieza, Kozienicki, Dolina Baryczy, Wdecki) across Poland in March and April 2017 (Fig. 1). Within each city, individuals were sampled in 3 distinct urban environments, namely the city centre, a residential area, and an urban park. For the city of Warsaw, those 3 habitats and the suburban forest have been additionally replicated three times. Birds were attracted to a mist-net using a loud-speaker playing calls from the two focal species as well as a dummy of a great tit. The birds were aged (1-year-old or older) and sexed based on their plumage features^43,44^. Additionally, from each bird, the second tail feather from the left side was plucked and stored in individual paper bags until MTE analyses. The protocol was performed in accordance with the Directive 2010/63/EU of the European Parliament and of the Council of 22 September 2010 on the protection of animals used for scientific purposes. Moreover, this study was approved by the Local Ethical Committee nr I for Animal Experimentation in Warsaw (I Lokalna Komisja Etyczna ds. Doświadczeń na Zwierzętach w Warszawie; permit no. 220/2016).

The feathers of 179 males (97 blue tits and 82 great tits) were analysed for their MTE content (see “MTE quantitative analysis” section), out of a larger dataset of 350 individuals (140 male and 56 female blue tits, and 111 male and 43 female great tits). Feather selection was based on two criteria: first, due to contrasted dispersal strategies (males disperse shorter distances from their natal or previous breeding site than females^45–48^) and because the sample size for females was too small to accurately test the link between MTE concentrations and urbanization level, we standardized our data set by selecting only males; those represented over 70% of the individuals that were sampled. The number of males caught varied substantially between habitats. Therefore, out of 251 males, a sub-sample of feathers from 179 males were selected in a way to maximise dataset balance in order to generate comparable sample sizes between species per habitat category (X^2^ = 1.44, df = 4, P = 0.838; Table D1) and age class per habitat category (X^2^ = 6.51, df = 4, P = 0.164); the samples were randomly selected before analysis.

### MTE quantitative analysis

Feathers were prepared for metallic/metalloid trace element (MTE) analyses using the protocol from our previous study^16^. The following MTEs were quantified: lead, zinc, copper, cadmium, arsenic, mercury. Briefly, feathers were washed alternatively with 0.25 M NaOH solution and ultrapure water (Milli-Q purified, Merck KGaA, Darmstadt, Germany) to remove external contamination, then dried 12 h at 50 °C to dry mass. Feathers were digested in 1 mL of HNO_3_ 30% for 24 h at 80 °C. The product of digestion was transferred into plastic tubes and ultrapure water was added to reach a final 1% acid concentration. Total content of lead (Pb; average of Pb 206, Pb 207 and Pb 208 isotope concentrations), zinc (Zn; average of Zn 66 and Zn 68 isotope concentrations), copper (Cu; average of Cu 63 and Cu 65 isotope concentrations), cadmium (Cd; Cd 111 concentrations), arsenic (As; As 75 concentrations) and mercury (Hg; average of Hg 200 and Hg 202 isotope concentrations) were determined using an inductively coupled plasma mass spectrometer (NexION 300D ICP Mass Spectrometer, Perkin Elmer SCIEX, USA). A conventional Mainhardt nebulizer and a quartz cyclonic spray chamber were used for sample introduction. Each isotope was measured three times and each sample was analysed two times. Relative standard deviation between the three measurement per isotope and between the two measurements per sample were all below 10%; no measurement was excluded. Quantification limits were as follows: Pb: 0.31 ppm, Zn: 1.32 ppb, Cu: 0.72 ppb, Cd: 0.045 ppb, As: 0.077 ppb and Hg: 0.065 ppb. The ICP-MS was calibrated before performing measurements with the use of multi standard solutions (ICP Calibration Standard from Merck). During the measurements, the parameters of calibration were checked using the standard containing mercury at the concentration of 1 µg/L in 1% nitric acid. The blanks and Certified Reference Materials (CRMs; trace elements in water 1643f from LGC Standards and SPS-SW1 batch 112 from SpectraPure Standards) were prepared and analysed using the same methods as the samples. The recovery of the CRMs ranged from 90 to 110%. Concentrations measured in the blank were extremely low: they were 1.401 ppb for zinc, 0.508 ppb for arsenic and below quantification limits for copper, lead, cadmium and mercury. All measurements were performed at the Biological and Chemical Research Centre (NCBCh, University of Warsaw, Poland). Correlations between each pair of MTEs are presented in Fig. A1.

### Quantifying urbanization

**Figure 2:**
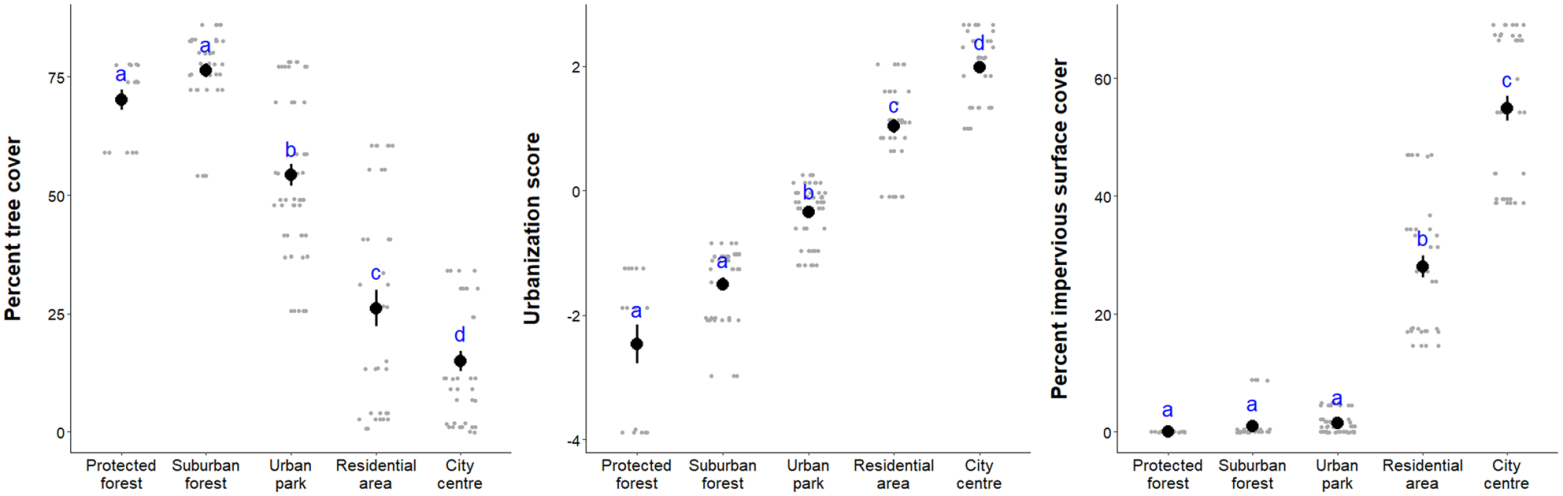
Mean ± se urbanization level, either percent tree cover, percent impervious surface cover or urbanization score (i.e. computed from imperviousness, tree cover, distance to the closest road and distance to the city centre) per habitat category. Significant differences of urbanization level between habitats are indicated by different letters.

Impervious surface cover and tree cover in a 100 m radius around each mist-net sampling point was extrapolated via satellite imagery following the method described in Ref.^19^. Briefly, tree cover (i.e. the percentage of trees) and impervious surface cover (i.e*.* the percentage of soil sealing and built-up areas) were downloaded from Copernicus Land Monitoring Services; the basic maps referred to 2015 and are of 20 m pixel resolution. Distance to the closest local road and to the city centre were calculated using GIS 2.8.2 and Google maps, respectively. For each city, the coordinates of the city centres were extracted from Wikipedia. Urbanization was quantified as (i) tree cover, (ii) impervious surface cover and (iii) an urbanization score computed from a Principal Component Analysis on the four environmental variables that were measured in this study: tree cover, impervious surface cover, distance to the closest road and distance to the city centre. Those three quantitative indexes of urbanization are commonly used in urban ecology^19,49,50^. Tree cover, distance to the closest road and distance to the city centre were all positively correlated, while impervious surface cover was negatively correlated with the other environmental variables (Fig. A2). Based on the Kaiser-Guttman criterion, one component, hereafter named “Urbanization score”, was retained in the PCA. It accounts for 61.4% of the variance in the data set; it is positively correlated to the impervious surface cover (r = 0.85) and negatively correlated to tree cover (r =  − 0.80), the distance to the closest road (r =  − 0.69) and the distance to the city centre (r =  − 0.68). While a composite multivariate metric (here urbanization score) is the most accurate index of urbanization, univariate metrics that are highly correlated to such multivariate metric (here tree cover and imperviousness surface cover) are preferred as they are unambiguous and readily comparable between studies^19^. In addition to the three quantitative indexes of urbanization listed above, we also categorized the environment where the birds have been caught into 5 habitats with contrasted environmental features and land use; when arranged from the lowest to the highest urbanization level, the habitats are ordered as follows: protected forest, suburban forest, urban park, residential area and city centre (Fig. 2).

### Statistical analyses

Statistical analyses were performed using R software (version 4.0.3)^51^. The percentage of data above the MTE quantification limit were similar between blue tits and great tits. Overall, they were 100% for Zn, 99% for Cu, 92% for Pb, 91% for As, 59% for Cd and 21% for Hg. Data below the quantification limits were given a value using a regression on order statistic (‘ROS’ function of the ‘NADA’ package); this estimation was done separately in blue tits and great tits^52^. To minimize the influence of possible spurious outliers on the distribution of MTE concentrations, values more than 1.5 times the interquartile range from the quartiles (i.e. either below Q1 − 1.5IQR, or above Q3 + 1.5IQR) were removed^53^; the procedure was done separately for blue tits and great tits on log-transformed values. In total, 16 values (ca. 1.5% of the values) were identified as outliers. Note that, although the output of the models slightly changed, the results are the same whether outliers were removed or not (see Table B1).

MTE concentrations in blue tit and great tit feathers were compared using linear mixed-effects models with MTE concentrations (i.e. Cu, Zn, Pb, As, Cd or Hg after log transformation) as the response variable and urbanization level (computed as either impervious surface cover, tree cover, urbanization score or habitat category) as the explanatory variable. MTE concentrations may vary differently in response to bird moulting pattern, foraging behaviour, seasonal movements and/or MTE metabolism. Those are known to differ between species and age^43,44,54–56^. Therefore, species, age, their interaction as well as their interactions with the urbanization level were added as explanatory variables and the single terms “species” and “age” were considered as categorical control variable. The location (i.e. either the city or the protected forest) was added as random intercept. Lmer were fitted with restricted maximum likelihood (REML) method using the ‘lme4’ package. Normality of model residuals was validated using quantile–quantile plots. For each model, we performed a backward stepwise selection using the AIC^57^. A Type III Wald Chi-square test Anova was used to determine the significance of retained variables in the final models. When discrete explanatory variables were retained in the models, contrasts among groups were tested using least-square mean pairwise comparisons (contrast function of the ‘lsmeans’ package)^58^.

The proportion of variance in MTE concentrations that is explained by urbanization level (computed either from tree cover, impervious surface cover or urbanization score) was calculated using the ‘r2_nakagawa’ function of the ‘performance’ package. It was calculated using two metrics of relative importance: (i) the difference between the conditional r-squared of the full model and the conditional r-squared of the model without the urbanization index as explanatory variable (defined as the “last” metric in the ‘relaimpo’ package) and (ii) the marginal r-squared of the model including the urbanization index only as explanatory variable (defined as the “first” metric in the ‘relaimpo’ package)^59^. The “last” and the “first” metrics tend to underestimate and overestimate, respectively, the variance explained by the variable of interest (here the urbanization index), meaning that the exact variance explained by the urbanization index falls between the two values computed from those two metrics^59^. To further investigate what environmental variable(s) better explain(s) the variation in MTE concentrations, the two metrics were also computed for the distance to the closest road and the distance to the city centre (Table D2).

## Results

### Variation in MTEs concentrations along continuous urbanization gradients

**Figure 3:**
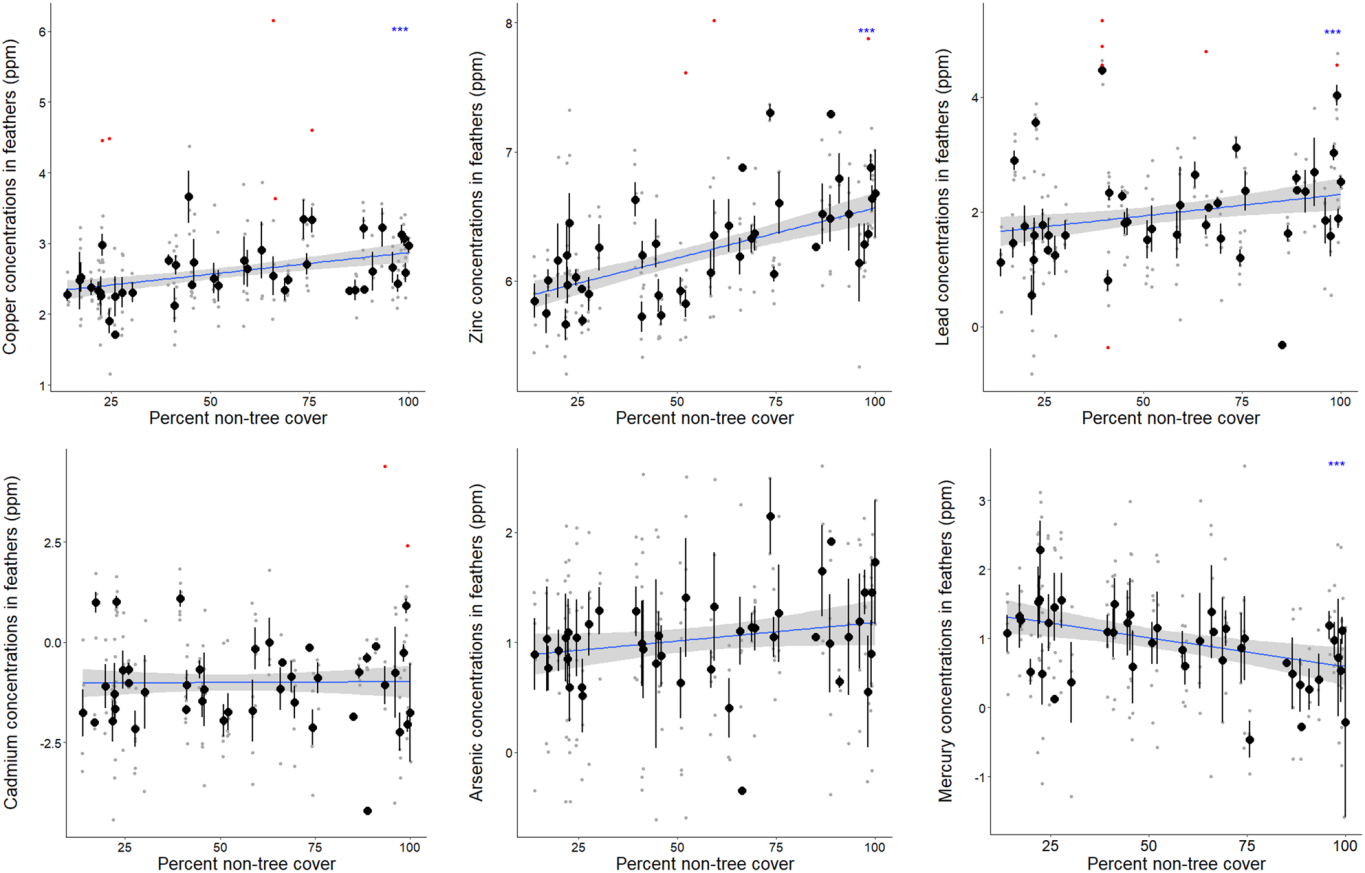
Relationship between MTE concentrations (i.e. Cu, Zn, Pb, Cd, As or Hg after log-transformation; in ppm) and the urbanization level (here tree cover). For the x axis to positively correlates with the urbanization level, we highlight the percent of non-tree cover (100—percent of tree cover). We highlight the concentration for each single individual (grey dots), the concentrations that were considered as outliers (in red), the mean ± se concentration per percent of non-tree cover (in black) and the regression line (in blue) and its confidence interval (in grey). The statistical significance of the relationship is highlighted with asterisks. Species-specific and age-specific relationships between MTE concentrations and the urbanization level are displayed in Figs. C1 and C2, respectively.

We measured a consistent association between urbanization level and the concentrations of several MTEs within the 8 replicated cities: whatever the species and age of the individuals, Cu, Zn and Pb increased while Hg decreased with increasing urbanization (i.e. with decreasing tree cover but with increasing impervious surface cover or urbanization score); Cd and As did not vary in response to urbanization (see Table 1, Fig. 3 for results on tree cover; the results of the models using impervious surface cover and urbanization score as a proxy of urbanization level are presented in Table D1). Urbanization level explained a maximum of 12% of Pb variation (Table 1) and 16%, 30%, and 8% of Cu, Zn and Hg variation, respectively (Table D1). Models including “distance to centre” or “distance to road” systematically fitted worse than the models including one of the urbanization indexes (Table C1). Moreover, MTE concentration in feathers are species- and age-specific: Cu, Pb and As were higher in the feathers of blue tits than of great tits (Table 1, Supplementary Table D1, Fig. C1). Pb and Zn were higher in the feathers of 1-year-old birds than of older birds, although the relationship was only marginally non-significant for Zn when considering urbanization score as proxy of urbanization level (Table 1, Supplementary Table D1, Fig. C2).

**Table 1:** Results of the best fitting statistical models testing the link between MTE concentrations (Cu, Zn, Pb, Cd, As and Hg) and urbanization level (here tree cover) while taking into account the species, the age and the location, tested in 8 cities and 4 protected forests.

	Cu	Zn	Pb	Cd	As	Hg
Tree cover	**Χ**^**2**^** = 24.79, P < 0.001, β = **− **0.006, 0.095 < r**^**2**^** < 0.117**	**Χ**^**2**^** = 70.53, P < 0.001, β = **− **0.009, 0.260 < r**^**2**^** < 0.260**	**Χ**^**2**^** = 40.06, P < 0.001, β = **− **0.012, 0.120 > r**^**2**^** > 0.103**			**Χ**^**2**^** = 11.55, P < 0.001, β = 0.009, 0.052 < r**^**2**^** < 0.057**
Species	**Χ**^**2**^** = 28.53, P < 0.001**	Χ^2^ = 2.80, P = 0.094	**Χ**^**2**^** = 7.55, P = 0.006**	Χ^2^ = 3.26, P = 0.071	**Χ**^**2**^** = 20.15, P < 0.001**	Χ^2^ = 0.21, P = 0.644
Age	Χ^2^ = 0.41, P = 0.52	Χ^2^ = 2.70, P = 0.100	**Χ**^**2**^** = 11.69, P < 0.001**	Χ^2^ = 1.50, P = 0.220	Χ^2^ = 2.34, P = 0.126	Χ^2^ = 1.78, P = 0.181
R^2^	0.351	0.410	0.584	0.353	0.108	0.052

Degrees of freedom were 1 for all the variables. The proportion of the variance in MTE concentrations that is explained by the urbanization level—r^2^—is comprised between two values computed from the metrics “first” and “last”^57^. For Hg, the random effect “city” had a zero variance, preventing to calculate the coefficient of determination—R^2^—of the model; for this reason, we report for Hg the adjusted coefficient of determination from the linear model.

Significant effects (P < 0.05) are highlighted in bold. Results are strikingly similar when using impervious surface cover and urbanization score as metric for urbanization quantification; these are reported in Table D1.

### Variation in MTE concentrations between habitat categories

**Figure 4:**
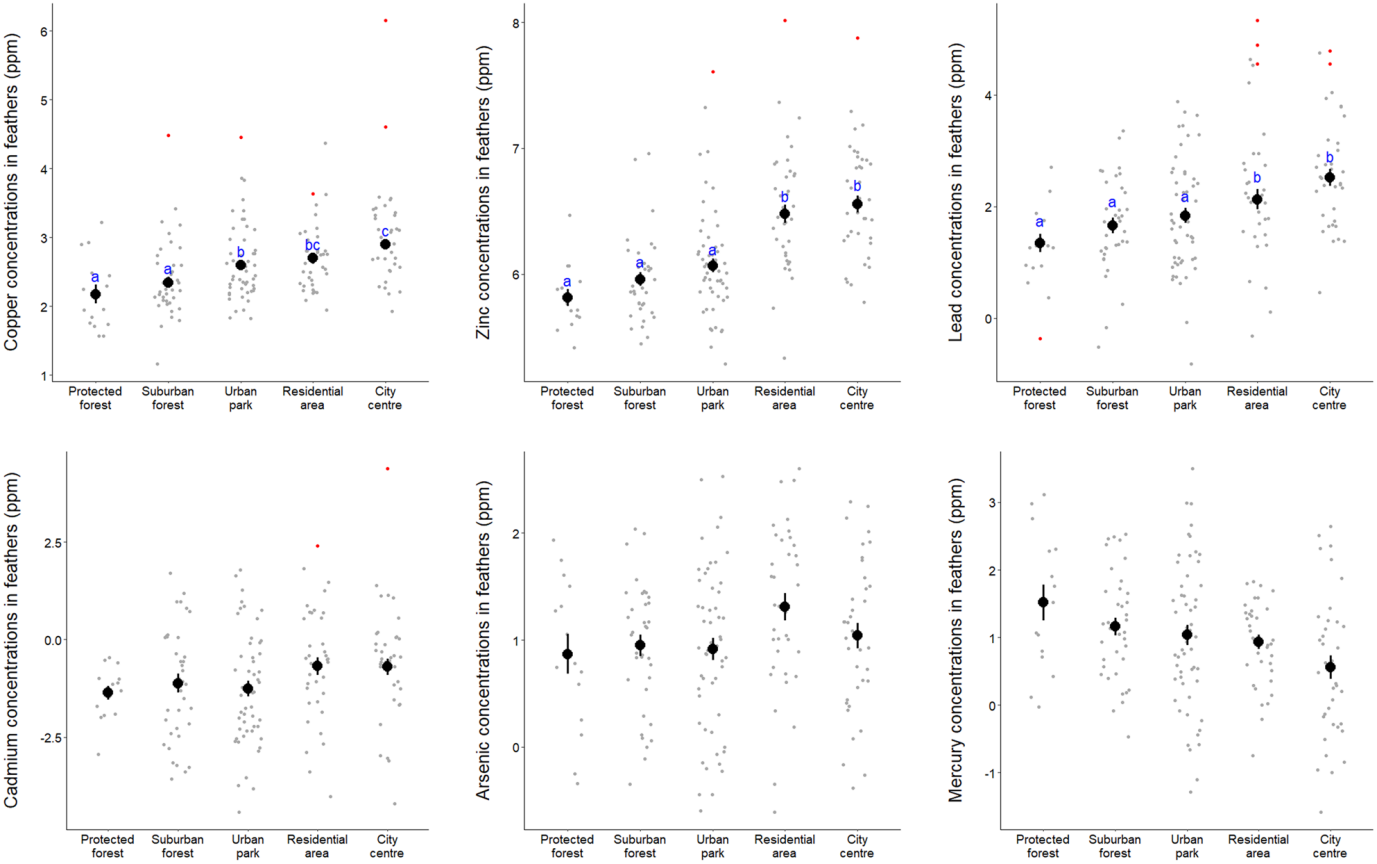
Mean ± se MTE concentrations (Cu, Zn, Pb, Cd, As or Hg after log-transformation; in ppm) per habitat category (protected forest, suburban forest, urban park, residential area or city centre). Significant differences of MTE concentrations between habitat categories are indicated by different letters.

Cu, Zn and Pb exhibited considerable variation from one habitat to another, and between urban and rural sites: overall, they were higher in city centres and residential areas than in urban parks, suburban forests, and protected forests (Table 2, Fig. 4). For instance, Cu (19.2 ppm), Zn (750.8 ppm) and Pb (28.1 ppm) in urban centres were ca. 74%, 82% and 135% higher than Cu (11.6 ppm), Zn (407.9 ppm) and Pb (7.1 ppm) in adjacent suburban forests, respectively (data between brackets are mean values). Similar to the previous models, Cu, Pb and As were higher in the feathers of blue tits than of great tits (Table 2) and Pb and Zn were higher in the feathers of 1-year-old birds than of older birds (Table 2). Raw MTE concentrations per species and per habitat are detailed in Table E1.

**Table 2:** Results of the best fitting statistical models testing the link between MTE concentrations (Cu, Zn, Pb, Cd, As and Hg) and the habitat category while taking into account the species, the age and the location.

	Cu	Zn	Pb	Cd	As	Hg
Habitat	**X**^**2**^** = 39.87, P < 0.001**	**X**^**2**^** = 92.33, P < 0.001**	**X**^**2**^** = 50.29, P < 0.001**			
Species	**X**^**2**^** = 30.92, P < 0.001**	X^2^ = 3.05, P = 0.081	**X**^**2**^** = 8.33, P < 0.001**	X^2^ = 3.26, P = 0.071	**X**^**2**^** = 20.15, P < 0.001**	X^2^ = 0.43, P = 0.512
Age	X^2^ = 1.17, P = 0.280	**X**^**2**^** = 3.97, P = 0.046**	**X**^**2**^** = 15.40, P < 0.001**	X^2^ = 1.50, P = 0.220	X^2^ = 2.34, P = 0.126	X^2^ = 0.80, P = 0.370
R^2^	0.387	0.420	0.590	0.332	0.121	≈ 0

Degrees of freedom were 4, 1 and 1 for habitat, species and age, respectively. Significant effects (P < 0.05) are highlighted in bold.

## Discussion

For the first time, we report on a highly significant and consistent positive linear relationship between the magnitude of urbanization—inferred as either impervious surface cover, the non-tree cover or an urbanization score computed from several environmental variables—and copper, zinc and lead concentrations in bird feathers sampled across 8 replicated urban–rural gradients; the reverse relationship was measured in the case of mercury. Importantly, those relationships were similar when quantified in two passerine species, and for both 1-year-old and older birds. Strikingly, the significant covariation between urbanization and specific metallic trace elements (MTEs) in avian feathers were measured across 8 replicated urbanization gradients, which included 8 cities, 8 suburban forests and 4 protected forests. This result confirms the pervasive impact on urbanization on the presence of MTEs in wild organisms, irrespective of the fact that the 8 cities differed in terms of size and population density (Fig. 1).

When arranged from the least to the most urbanized site, habitat categories were ordered as follows: protected forest, suburban forest, urban park, residential area, and city centre (Fig. 2). Therefore, it is not surprising that we measured higher concentrations of copper, zinc and lead in the feathers of the birds sampled at the most urbanized habitats than in the feathers from birds caught in the less urbanized habitats. Interestingly, zinc and lead concentrations measured in individuals sampled in urban parks were not significantly different from the concentrations measured in their conspecifics sampled in suburban or protected forests. This result, as well as the fact that urban parks are the least urbanized urban habitats, are a striking demonstration that comparing bird populations between urban parks and forests, a still common study design in urban ecology^19^, is not appropriate to study (i) how MTE exposure varies in response to urbanization and (ii) the ecology and evolution of animal populations.

Importantly, the strength and direction of the association between MTE concentrations and urbanization level varied between MTEs. Indeed, copper, zinc and lead concentrations increased, while mercury concentrations decreased along the rural–urban gradient; cadmium and arsenic concentrations did not significantly vary along such a gradient. Those results suggest that copper, zinc and lead are mainly emitted by anthropogenic activities occurring within urban areas. The fact that the concentrations of those three MTEs were highly correlated (the correlation coefficient ranged between 0.38 and 0.65) also suggests that those MTEs share common emission source(s). Therefore, and although this study did not aim at identifying the source of such pollutants, our results tend to hold road traffic responsible for most of urban-driven copper, zinc and lead emissions (through past leaded gasoline combustion, tire wear and brake pad abrasion and other vehicle emissions)^28^. On the contrary, our results suggest that mercury emissions are higher outside of cities, where they may stem from coal burning, caustic soda and/or cement production^60^. Finally, the activities responsible for cadmium and arsenic emissions (e.g. steel, plastic and pigment production, coal combustion^28^), may be distributed more homogenously along the urban–rural gradient, which would explain the lack of linear association between the concentrations of those two MTEs and the urbanization level.

It is noteworthy that, in our study, the birds were caught at the beginning of their reproductive season (i.e. in March and April). Yet, because feathers were washed to removed most external contamination^35^, MTE concentrations in the feathers mainly reflect the concentrations of those same MTEs in the environment where the birds moulted (i.e. between July and September), which is the nest where they hatched (if first-year breeders), or their previous breeding ground (for 2nd-year breeders or older) in most bird species with seasonal moulting, including the blue tit and the great tit^43^. Therefore, the associations measured between MTE concentrations and urbanization level might have been weakened by some dispersal events along the urban–rural gradient. In other words, these associations might have been stronger if the birds were caught at the place where they grew their feathers. On the other hand, the fact that we did measure significant associations between copper, zinc, lead and mercury concentrations and the urbanization gradient suggests that male blue tits and great tits have limited movements along the urban–rural gradient. While this interpretation should be confirmed using a capture-mark-recapture or a radiotracking approach, the concentrations of some MTEs (especially zinc) in bird feathers appears like a promising tool to gain insight into natal and breeding dispersal along the urban–rural gradient^61^.

While the association between MTE concentrations and urbanization level were equivalent in both tit species and in both age categories, blue tits exhibited higher levels of copper, lead and arsenic than great tits. Similarly, previous studies also measured higher levels of MTEs in the feathers of blue tits than of great tits^16,62,63^ (but see^64^). Such a difference may result from differences in diet, metabolism or MTE transfer into the feathers, the latest depending on the concentrations of keratin and melanin in the feather^65,66^. For instance, previous studies suggest that great tits and blue tits differ in regard to their exposure, metabolism and/or need of calcium^24,67^. Yet, calcium downregulates lead and cadmium absorption in birds^68–70^. Further investigation on the underlying mechanisms explaining the higher levels of MTEs in blue tits than in great tits are needed to understand whether the blue tit is likely to show a higher response to urban MTE pollution. Our study also shows that younger birds have higher concentrations of lead, and to a lower extent zinc, than older birds. Similarly, experimental studies on laboratory animals and data on humans showed that lead concentrations are higher in young than in older individuals as a result of age-related lead gastrointestinal absorption rate^71^. This result suggests that young birds are more likely to respond to urban MTE lead pollution than older ones. In line with this hypothesis, several studies demonstrated the deleterious effects of urban lead pollution on nestlings^16,26,72^. Finally, we should highlight the fact that this study measured MTE concentrations in males only; although previous studies measured only weak gender-related differences in MTE concentrations in feathers^63,64^, we cannot exclude that MTE exposure and metabolism may vary between the sexes. Therefore, further studies are needed to generalize our results to both sexes.

Importantly, the levels of lead measured in individuals sampled in more urbanized areas (19.6 ppm in individuals caught in city centres) were more than two times higher than the levels shown to induce reproductive impairments or to alter immunity in other bird species^26,72,73^. Altogether, those results suggest that the effects of urban copper, zinc and lead pollution on birds (e.g., on their survival, reproductive success, immunity, corticosterone levels and plumage colouration^15,16,26,72–75^) are expected to increase from less to more urbanized environments. In line with this prediction, rates of phenotypic change are greater in urban environments compared with non-urban landscapes^2^ and numerous studies measured phenotypic divergence between urban and rural populations^3^. Consequently, we can hypothesize that some of those urban-driven phenotypic changes (e.g., oxidative balance, xenobiotic metabolism, immunity, gene expression) may be triggered by urban MTE pollution. For instance, in the great tit, genes involved in antioxidant defences are expressed at a higher rate in individuals sampled within the city of Malmö (Sweden) than in their counterparts sampled in an adjacent forest; those individuals also exhibited a higher expression of the genes involved in xenobiotic metabolism^76^. Similar patterns were measured in the white-footed mouse (*Peromyscus leucopus*)^77^. The toxicity of several MTEs, including lead, resulting partly from the fact that they induce oxidative damages^78^, suggests that urban MTE exposure may trigger such modification in gene expression levels^79^.

Phenotypic differences can result from adaptive or non-adaptive adjustments^1,80,81^. As the exposure to urban MTEs is often associated with detrimental effects on bird fitness^16,26,72^, urban MTE pollution is likely to exert selective pressures on populations inhabiting more urbanized environments. Importantly, this study reveals that urban MTE-triggered selective pressure is likely to be directional and consistent across a large number of urban sites, and thereby conducive to the emergence of parallel responses at the genetic and/or phenotypic level^17^. As a matter of fact, there is some evidence that more melanistic individuals transfer higher amounts of MTEs in their teguments (i.e. feathers and skin)^72,82^ and are positively selected in environments polluted with MTEs^72,82–84^. Consequently, comparing phenotypic traits (e.g., tegument melanin-based colouration, MTE gastro-intestinal absorption rates, antioxidative response, metallothionein expression) and allele frequency at loci associated with the metabolism of MTEs (*e.g., GCLC*, *GCLM* and *MT2A* genes)^85,86^ along replicated urbanization gradients appears as a promising opportunity to study parallel evolution mediated by urbanization^17^.

## Supplementary Information

Supplementary Information.

## Data Availability

Data are available on GitHub at https://github.com/MarionChatelain/10.1038-s41598-021-99329-2.
