# Author Correction: Replicated, urban-driven exposure to metallic trace elements in two passerines

**DOI:** 10.1038/s41598-021-99964-9

**Published:** 2021-10-08

**Authors:** Marion Chatelain, Arnaud Da Silva, Marta Celej, Eliza Kurek, Ewa Bulska, Michela Corsini, Marta Szulkin

**Affiliations:** 1 Centre of New Technologies, University of Warsaw, 02-097 Warsaw, Poland; 2 Department of Zoology, University of Innsbruck, Technikerstraße 25, 6020 Innsbruck, Austria; 3 Faculty of Chemistry, Biological and Chemical Research Centre, University of Warsaw, 02-089 Warsaw, Poland

Correction to: *Scientific Reports* https://doi.org/10.1038/s41598-021-99329-2, published online 04 October 2021.

The Data Availability section in the original version of this Article was omitted.

It now appears as below:

"Data are available on GitHub at https://github.com/MarionChatelain/10.1038-s41598-021-99329-2".

The original Article has been corrected.

