# Supplementary material for: Replicated, urban-driven exposure to metallic trace elements in two passerines
Source: Sci Rep. 2021 Oct 4;11:19662. doi: 10.1038/s41598-021-99329-2 (PMC8490372; doi:10.1038/s41598-021-99329-2)
Supplement: Supplementary file 1 — Supplementary Information. [file 41598_2021_99329_MOESM1_ESM.pdf]

Replicated, urban-driven exposure to metallic trace elements in two passerines

Marion Chatelain[1,2,*], Arnaud Da Silva[1], Marta Celej[1], Eliza Kurek[3], Ewa. Bulska[1,3], Michela Corsini[1], Marta Szulkin[1]

[1] Centre of New Technologies, University of Warsaw, 02-097 Warsaw, Poland

[2] Department of Zoology, University of Innsbruck, 6020 Innsbruck, Austria

[3] Faculty of Chemistry, Biological and Chemical Research Centre, University of Warsaw, 02-089 Warsaw, Poland

*Correspondence: Department of Zoology, University of Innsbruck, Technikerstraße 25, 6020 Innsbruck, Austria, marion.chatelain@uibk.ac.at, +43 (0)512 507-51866

Appendix A

Figure A1. Correlations between MTE concentrations (*i.e.* Cu, Zn, Pb, As, Cd and Hg) in the feathers of blue tits (BT) and of great tits (GT). Scatterplots of each pair of numeric variables are drawn on the left part of the figure. Pearson correlations are displayed on the right; ; they were calculated using the 'ggpairs' function of the 'GGally' package. Variable distribution is available on the diagonal.

***P≤0.001 **P≤0.01 *P≤0.05 ·P≤0.10

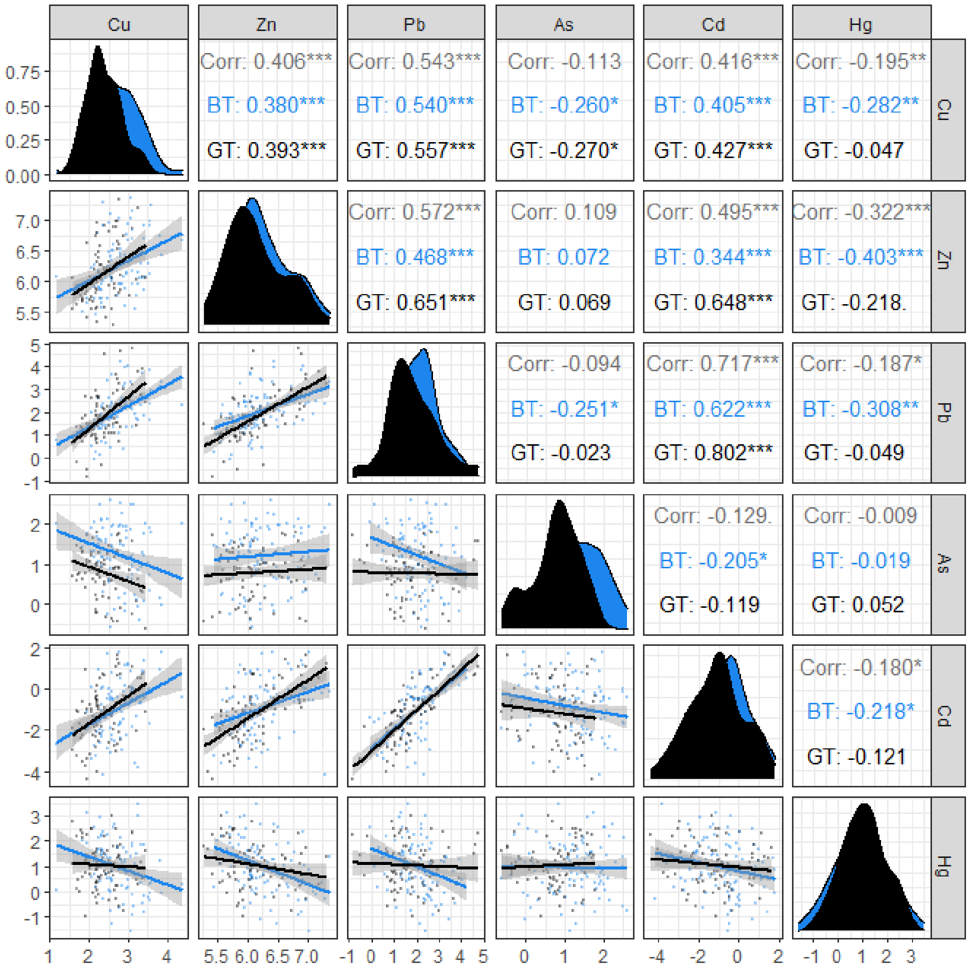

Figure A2. Correlations between the four environmental variables measured in this study (*i.e.* imperviousness, tree cover, distance to the closest road, distance to the city centre). Scatterplots of each pair of numeric variables are drawn on the left part of the figure. Pearson correlations are displayed on the right; they were calculated using the 'ggpairs' function of the 'GGally' package. Variable distribution is available on the diagonal.

***P≤0.001 **P≤0.01 *P≤0.05 ·P≤0.10

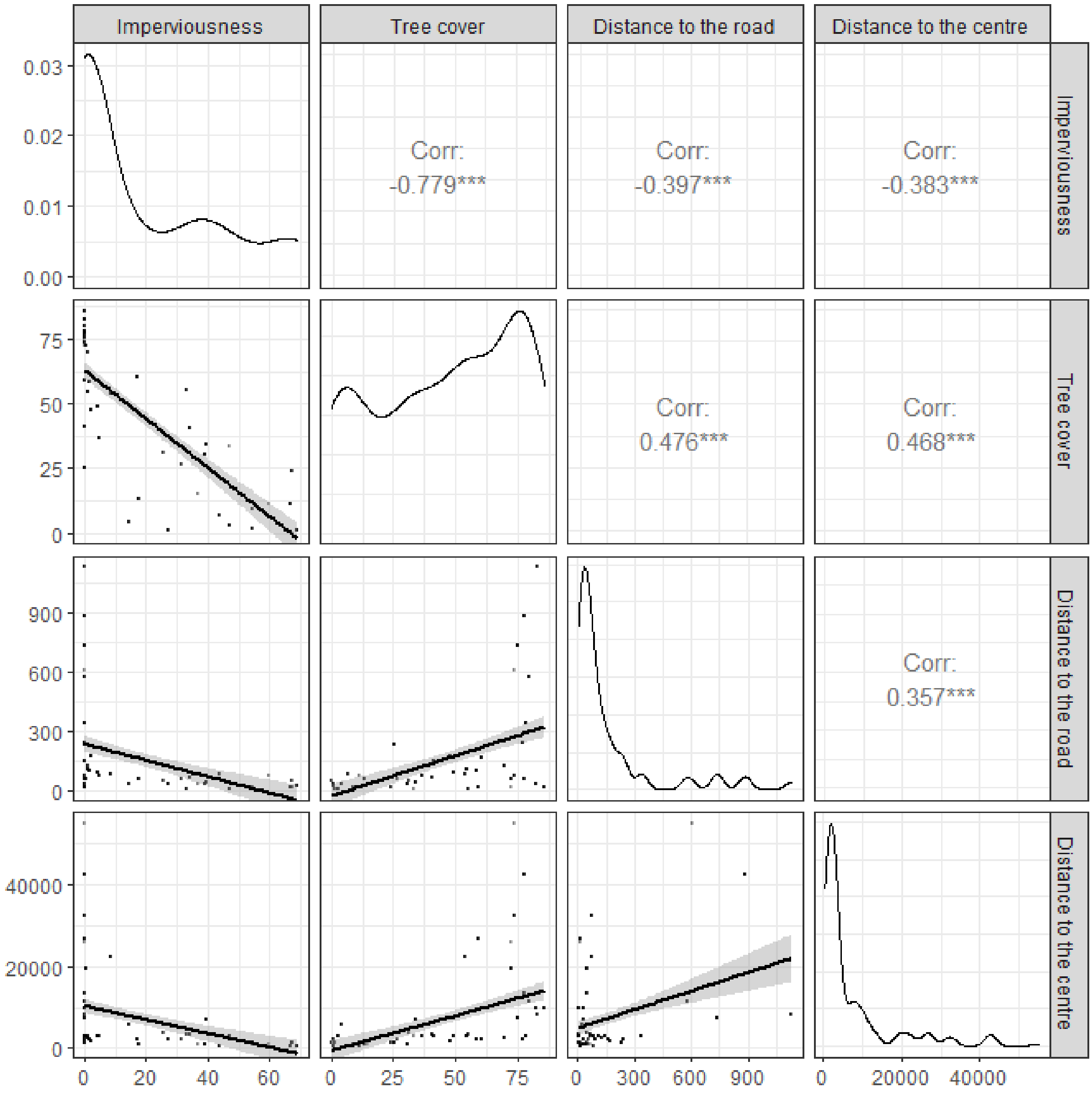

Appendix B

Table B1. Results of the best fitting statistical models testing the link between MTE concentrations (Cu, Zn, Pb, Cd, As and Hg) and urbanization level (here tree cover) while taking into account the species and the age. The models were run on the complete data set, without excluding outliers. The proportion of the variance in MTE concentrations that is explained by urbanization score or impervious surface cover – r^2 – is comprised between two values computed from the metrics "first" and "last" [31].

	**Cu**	**Zn**	**Pb**	**Cd**	**As**	**Hg**
Tree cover	X^2 = 14.30, P < 0.001, β = -0.006, 0.060 < r^2 < 0.186	X^2 = 65.11, P < 0.001, β = -0.009, 0.258 > r^2 > 0.248	X^2 = 34.61, P < 0.001, β = -0.012, 0.092 > r^2 > 0.087			X^2 = 11.55, P < 0.001, β = 0.009, 0.052 < r^2 < 0.057
Species	X^2 = 27.26, P < 0.001	X^2 = 2.77, P = 0.096	X^2 = 7.88, P = 0.005	X^2 = 2.90, P = 0.088	X^2 = 20.15, P < 0.001	X^2 = 0.21, P = 0.644
Age	X^2 = 0.33, P = 0.56	X^2 = 3.06, P = 0.080	X^2 = 9.91, P = 0.002	X^2 = 2.28, P = 0.131	X^2 = 2.34, P = 0.126	X^2 = 1.78, P = 0.181
R^2	0.277	0.374	0.583	0.279	0.121	0.052

Appendix C

Figure C1. Relationship between MTE concentrations (*i.e.* Cu, Zn, Pb, Cd, As or Hg after log-transformation; in ppm) and the urbanization level (here tree cover) in blue tits (in blue) and in great tits (in black). For the x axis to positively correlates with the urbanization level, we highlight the percent of non-tree cover (100 – percent of tree cover). We highlight the concentration for each single individual (grey dots), the mean ± se concentration per percent of non-tree cover (in black) and the regression line (in blue) and its confidence interval (in grey).

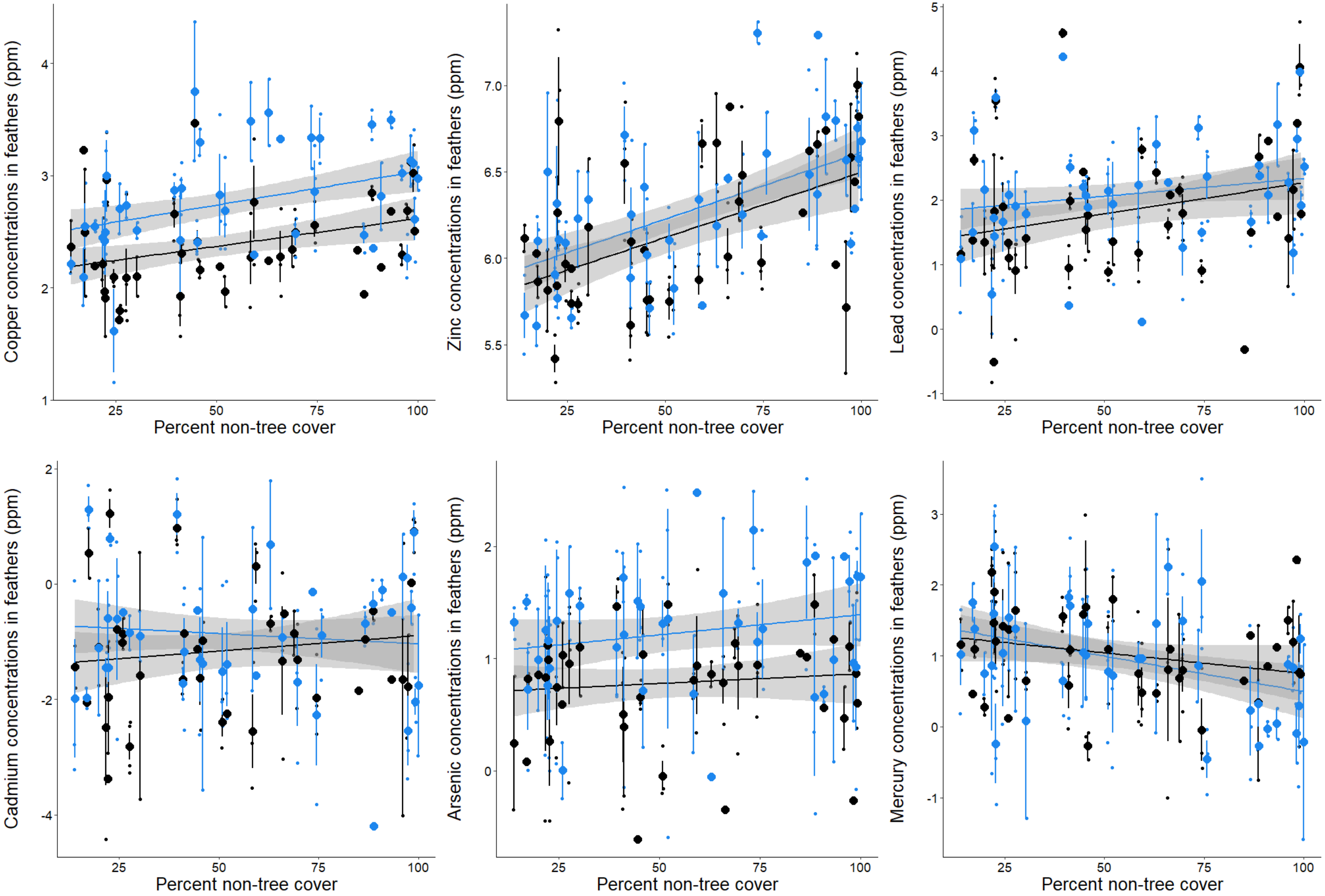

Copper concentrations in feathers (ppm)
Percent non-tree cover
Zinc concentrations in feathers (ppm)
Percent non-tree cover
Lead concentrations in feathers (ppm)
Percent non-tree cover
Cadmium concentrations in feathers (ppm)
Percent non-tree cover
Arsenic concentrations in feathers (ppm)
Percent non-tree cover
Mercury concentrations in feathers (ppm)
Percent non-tree cover

Figure C2. Relationship between MTE concentrations (*i.e.* Cu, Zn, Pb, Cd, As or Hg after log-transformation; in ppm) and the urbanization level (here tree cover) in one-year-old (in blue) and older individuals (in black). For the x axis to positively correlates with the urbanization level, we highlight the percent of non-tree cover (100 – percent of tree cover). We highlight the concentration for each single individual (grey dots), the mean ± se concentration per percent of non-tree cover (in black) and the regression line (in blue) and its confidence interval (in grey).

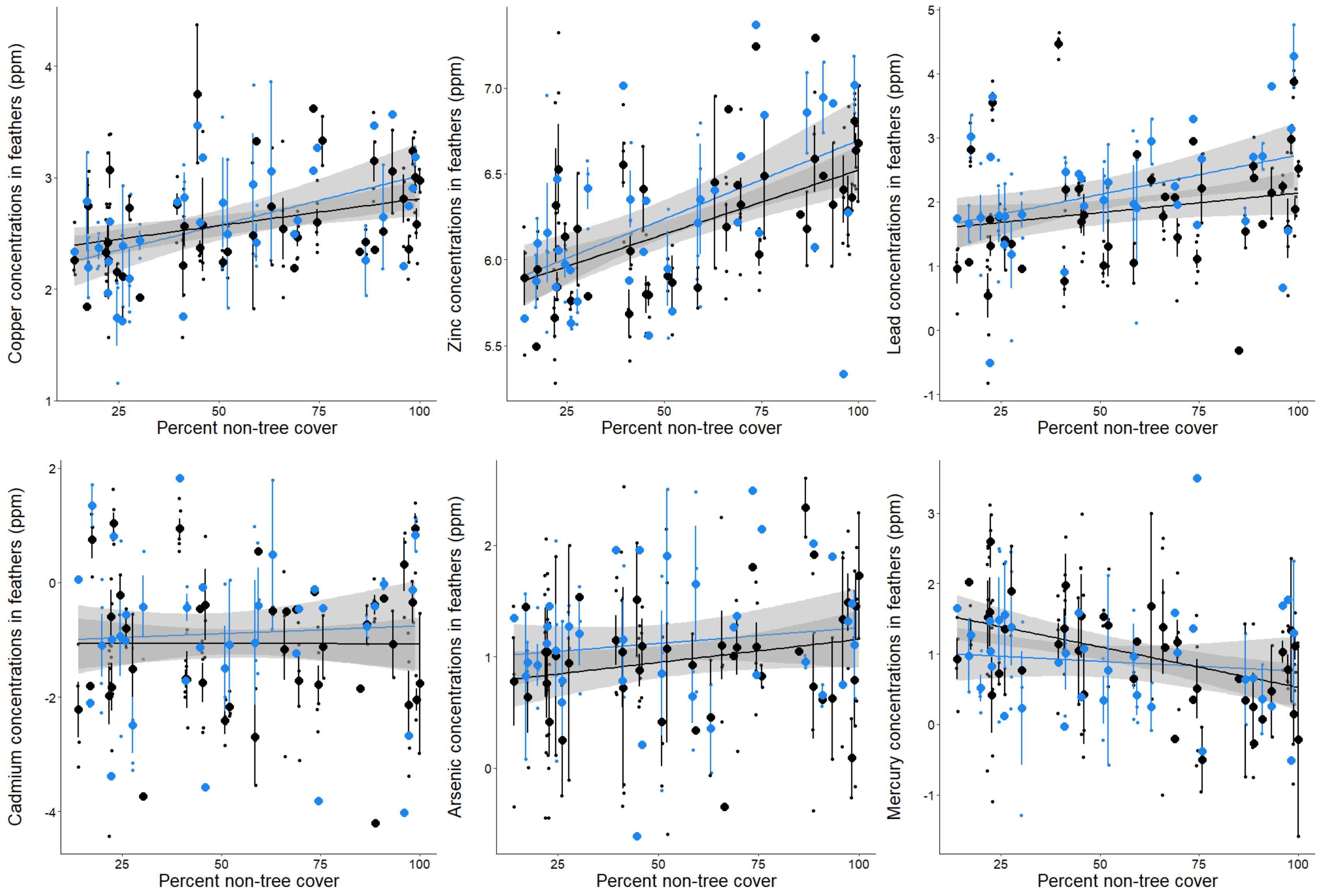

Copper concentrations in feathers (ppm)
Percent non-tree cover
Zinc concentrations in feathers (ppm)
Percent non-tree cover
Lead concentrations in feathers (ppm)
Percent non-tree cover
Cadmium concentrations in feathers (ppm)
Percent non-tree cover
Arsenic concentrations in feathers (ppm)
Percent non-tree cover
Mercury concentrations in feathers (ppm)
Percent non-tree cover
25
50
75
100

Appendix D

Table D1. Results of the best fitting statistical models testing the link between MTE concentrations (Cu, Zn, Pb, Cd, As and Hg) and urbanization score (a) or impervious surface cover (b) while taking into account the species and the age. The proportion of the variance in MTE concentrations that is explained by urbanization score or impervious surface cover – r^2 – is comprised between two values computed from the metrics "first" and "last". For Hg, the random effect "city" had a zero variance, preventing to calculate the coefficient of determination – R^2 – of the model; for this reason, we report for Hg the adjusted coefficient of determination from the linear model.

(a)	**Cu**	**Zn**	**Pb**	**Cd**	**As**	**Hg**
Urban. score	$X^2 = 33.49$, $P < 0.001$, $\beta = 0.128$, $0.096 < r^2 < 0.163$	$X^2 = 75.14$, $P < 0.001$, $\beta = 0.167$, $0.259 < r^2 < 0.295$	$X^2 = 41.13$, $P < 0.001$, $\beta = 0.239$, $0.112 < r^2 < 0.122$			$X^2 = 17.47$, $P < 0.001$, $\beta = -0.189$, $0.081 < r^2 < 0.084$
Species	$X^2 = 29.07$, $P < 0.001$	$X^2 = 2.73$, $P = 0.098$	$X^2 = 7.56$, $P = 0.006$	$X^2 = 3.26$, $P = 0.071$	$X^2 = 20.15$, $P < 0.001$	$X^2 = 0.15$, $P = 0.697$
Age	$X^2 = 0.74$, $P = 0.39$	$X^2 = 3.65$, $P = 0.056$	$X^2 = 13.45$, $P < 0.001$	$X^2 = 1.50$, $P = 0.220$	$X^2 = 2.34$, $P = 0.126$	$X^2 = 2.29$, $P = 0.130$
R^2	0.352	0.409	0.576	0.345	0.128	0.081

(b)	Cu	Zn	Pb	Cd	As	Hg
Impervious surface cover	$X^2 = 27.60$, $P < 0.001$, $\beta = 0.008$, $0.069 < r^2 < 0.129$	$X^2 = 70.84$, $P < 0.001$, $\beta = 0.011$, $0.201 < r^2 < 0.274$	$X^2 = 35.97$, $P < 0.001$, $\beta = 0.014$, $0.070 < r^2 < 0.098$			$X^2 = 15.64$, $P < 0.001$, $\beta = -0.012$, $0.073 < r^2 < 0.077$
Species	$X^2 = 28.58$, $P < 0.001$	$X^2 = 2.47$, $P = 0.116$	$X^2 = 7.54$, $P = 0.007$	$X^2 = 3.26$, $P = 0.071$	$X^2 = 20.15$, $P < 0.001$	$X^2 = 0.14$, $P = 0.712$
Age	$X^2 = 0.50$, $P = 0.48$	$X^2 = 2.55$, $P = 0.111$	$X^2 = 11.27$, $P < 0.001$	$X^2 = 1.50$, $P = 0.220$	$X^2 = 2.34$, $P = 0.126$	$X^2 = 1.94$, $P = 0.164$
R^2	0.325	0.351	0.534	0.337	0.129	0.073

Table D2. The proportion of the variance in MTE concentrations that is explained by distance to the closest road and distance to the city centre – r^2 – is comprised between two values computed from the metrics “first” and “last”.

	**Cu**	**Zn**	**Pb**	**Cd**	**As**	**Hg**
R^2 Distance to road	$0.011 < r^2 < 0.045$	$0.071 > r^2 > 0.062$	$0.034 > r^2 > 0.025$			$0.019 < r^2 < 0.026$
R^2 Distance to centre	$0.061 < r^2 < 0.121$	$0.070 < r^2 < 0.144$	$0.055 < r^2 < 0.083$			$0.041 < r^2 < 0.050$

Appendix E

Table E1. Sample size (N) and mean ± se MTE concentrations in the feathers (Cu, Zn, Pb, As, Cd, As and Hg; in ppm) per species (blue tit *i.e.* BT, or great tit *i.e.* GT) and habitat category (protected forest, suburban forest, urban park, residential area or city centre).

		**Protected forest**	**Suburban forest**	**Urban park**	**Residential area**	**City centre**
N	*BT*	*7*	*20*	*28*	*19*	*23*
	GT	*8*	*17*	*26*	*17*	*14*
**Cu**	BT	14.0 ± 2.6	12.7 ± 1.5	19.1 ± 2.0	19.6 ± 3.4	23.1 ± 1.9
	GT	6.7 ± 0.9	10.5 ± 1.4	11.2 ± 0.9	14.3 ± 1.7	15.2 ± 1.5
**Zn**	BT	324.6 ± 20.7	455.4 ± 50.0	470.2 ± 25.7	748.7 ± 81.2	783.4 ± 64.1
	GT	364.5 ± 46.4	360.3 ± 16.0	468.5 ± 59.9	666.3 ± 60.0	718.1 ± 78.0
**Pb**	BT	5.1 ± 1.2	8.6 ± 1.6	12.1 ± 2.0	11.9 ± 3.8	16.9 ± 3.3
	GT	4.4± 1.5	5.6 ± 1.1	8.0 ± 2.2	20.3 ± 7.8	22.3 ± 8.0
**Cd**	BT	0.3 ± 0.1	1.1 ± 0.3	0.9 ± 0.2	1.0 ± 0.4	0.8 ± 0.2
	GT	0.3 ± 0.1	0.5 ± 0.2	0.7 ± 0.2	1.1 ± 0.3	1.0 ± 0.3
**As**	BT	3.6 ± 1.0	3.5 ± 0.4	4.2 ± 0.6	6.5 ± 0.8	4.1 ± 0.6
	GT	2.4 ± 0.3	2.5 ± 0.3	2.4 ± 0.4	2.8 ± 0.4	2.8 ± 0.4
**Hg**	BT	10.2 ± 3.0	4.7 ± 0.8	5.5 ± 1.4	2.6 ± 0.3	2.5 ± 0.7
	GT	4.5 ± 1.9	3.8 ± 0.8	4.5 ± 0.9	3.4 ± 0.4	3.9 ± 1.1
